# Mammalian oogenesis and female reproductive aging

**DOI:** 10.18632/aging.101381

**Published:** 2018-02-05

**Authors:** Francesca E. Duncan, Jennifer L. Gerton

**Affiliations:** 1 Department of Obstetrics and Gynecology, Feinberg School of Medicine, Northwestern University, Chicago, IL 60611, USA; 2 Stowers Institute for Medical Research, Kansas City, MO 64110, USA; 3 Department of Biochemistry and Molecular Biology, University of Kansas Medical Center, Kansas City, KS 66160, USA

**Keywords:** advanced reproductive age, oocyte, aneuploidy, nucleolus, fibrosis

The female reproductive system is unique because it is the first to age in mammals, occurring decades prior to the deterioration of general organ function with age. In humans, female reproductive aging is characterized by a marked decline in reproductive function due to the loss of both the quantity and quality of gametes in the ovary, which begins when women enter their fourth decade and is complete by menopause. Due to these changes, women of advanced reproductive age have an increased risk of infertility, miscarriages, and birth defects. Reproductive aging is a robust biological phenomenon that will affect every single female regardless of her ethnicity, race, and geography. In fact, reproductive aging and its sequelae are becoming a significant health issue as more women globally are delaying childbearing and life expectancy is increasing.

The age-associated decline in fertility (maternal age effect) is attributed to the combined effects of reduced gamete quantity and quality. However, a recent study of women attempting to conceive naturally found that diminished markers of follicle quantity were not associated with infertility [1]. These findings strongly suggest that gamete quality, not quantity, is the prime factor in the reproductive age-associated decline in fertility. Clinical data from Assisted Reproductive Technology (ART) cycles further supports this notion. For example, when women use their own eggs to conceive via ART procedures such as in vitro fertilization, the likelihood of having a live offspring following embryo transfer decreases with age. In contrast, if women use eggs from young, healthy donors to conceive, the maternal age effect is abrogated. Therefore, the biological age of the egg dictates fertility outcomes, and gamete quality is a major component of the age-associated decline in fertility.

This knowledge has prompted a surge of research into the etiology of the age-associated decline in gamete quality which has highlighted the multifactorial nature of aging. A prime contributor to decreased gamete quality with age is aneuploidy or incorrect chromosome segregation during meiosis [2]. Oocyte meiosis is the process by which female germ cells undergo two rounds of cell division without an intervening round of DNA replication to ultimately produce a haploid gamete that upon fertilization will produce a diploid zygote. Oocyte meiosis is surprisingly highly error prone, and aneuploidy increases unequivocally and often exponentially with age [2]. The meiotic origins of aneuploidy are numerous, complex, and inter-related. For example, alterations in recombination sites have been associated with aging and aneuploidy [2]. Moreover, changes in chromosome associated proteins, such as cohesins and those at the kinetochore, can drive chromosome segregation errors with age [2]. These meiotic contributions to the aging-related decline in gamete quality may be caused or compounded by additional cytoplasmic changes. For example, mitochondria – the energy producing organelles of the cell - have altered morphology, increased mitochondrial DNA damage, and reduced function with age. More recently, meiotic non-disjunction that occurs with advanced reproductive age has been attributed to cytoplasmic defects in the microtubule cytoskeleton [3].

Many studies that investigate the age-related changes in the female gamete have focused on the end product – either fully grown oocytes from large antral follicles or mature metaphase II-arrested eggs that are obtained following in vitro maturation or ovulation. The period of oocyte growth which takes place during oogenesis is often overlooked because it is technically challenging to isolate follicles from the ovarian stroma especially in reproductively adult and aged mice. Moreover, it is difficult to query the oocyte in isolation from the follicle unit at this stage of development. However, oogenesis is a critical developmental window for gamete quality because the oocyte accumulates essential maternal products to drive the later events of meiotic maturation, fertilization, and early preimplantation embryo development. Oogenesis takes place over the course of weeks to months in mouse and human, respectively, and during this time, the germ cell undergoes tremendous volumetric expansion and is one of the most transcriptionally and translationally active cells in the body. Defects that occur during the oocyte growth phase will undoubtedly impact the quality of the resulting gamete. Therefore, in a recently published study, we performed RNA-Seq of individual growing follicles from reproductively young and old mice, followed by an in silico analysis to examine the aging gene expression signature in the oocyte and somatic cells during the active growth phase of oogenesis [4]. We identified differentially expressed genes that separated follicles by age, and importantly, genes related to mitochondria, chromosomes, kinetochores, microtubules, and spindles were all enriched in growing oocytes from reproductively young mice [4]. These findings are paradigm-shifting because they demonstrate that changes in the usual reproductive aging suspects occur very early in the developmental trajectory of the oocyte.

In addition, our data - for the first time - revealed protein homeostasis as a biological process that undergoes significant age-related changes during the oocyte’s growth phase and highlighted the nucleolus as a critical organelle in the aging process. We observed that, relative to young counterparts, oocyte nucleoli from reproductively old mice are present in greater numbers and have larger giant fibrillar centers, structures associated with active sites of ribosomal DNA transcription. In addition, oocyte nucleoli exhibit an age-associated increase in expression of fibrillarin, a ribosomal RNA methyltransferase that could alter ribosome function. These nucleolar changes are accompanied by an age-associated increase in ribosome numbers. Notably, similar nucleolar findings were observed in accelerated and physiologic aging in humans. For example, fibroblasts from patients with a premature aging disorder (progeria) exhibit nucleolar expansion and enhanced ribosome biogenesis, and this same phenotype was also observed in human fibroblasts across a physiologic aging spectrum [5]. Conversely, small nucleoli are associated with longevity, and knockdown of fibrillarin in *C. elegans* reduces nucleolar size and extends lifespan [6]. Thus, nucleolar alterations are a highly conserved hallmark of aging across tissues and organisms and warrant further investigation in the context of reproductive aging.

Not only do the earliest stages of oogenesis have a large impact on the age-related changes in gamete quality, but so does the microenvironment in which the gametes develop. We have shown previously that reproductive aging is associated with prominent fibrosis, macrophage infiltration, and inflammation in the ovarian stroma. Interestingly, our RNA-Seq data demonstrate that follicles from mice of advanced reproductive age express a preponderance of immune and inflammatory genes – mirroring what is occurring in the ovarian stroma [7]. Thus we speculate that there is close communication and integration between the follicles and their microenvironment, which likely influences the quality of the growing oocyte during oogenesis. The direct impact of the microenvironment on the follicle and oocyte is currently under investigation.

Taken together, it is clear that the age-associated deterioration of gamete quality is multifactorial and thus a wholistic perspective that considers all developmental stages and niches of the oocyte is required to fully understand underlying aging mechanisms. Unfortunately, there are inherent challenges to working with physiologic animal models of reproductive aging because of the time and cost it takes to age animals and the limited number of gametes that can be obtained per aged animal. Nevertheless, advances are constantly being made in the technologies that can be applied to single cells and limited biological material. Unbiased methods will be essential to unraveling the multifactorial origins of female reproductive aging.
